# Effect of Mobile Phone Use on Musculoskeletal Complaints: Insights From Nursing Students at Northern Border University, Arar, Saudi Arabia

**DOI:** 10.7759/cureus.57181

**Published:** 2024-03-29

**Authors:** Fathia Ahmed Mersal, Lobna Mohamed Mohamed Abu Negm, Manal S Fawzy, Ajitha Thankarajan Rajennal, Rehab Salamah Alanazi, Lujain Obaid Alanazi

**Affiliations:** 1 Department of Public Health Nursing, Faculty of Nursing, Northern Border University, Arar, SAU; 2 Department of Emergency Nursing, Faculty of Nursing, Northern Border University, Arar, SAU; 3 Unit of Medical Research and Graduate Studies, Northern Border University, Arar, SAU; 4 Department of Biochemistry, Northern Border University, Arar, SAU; 5 Department of Medical Surgical Nursing, Faculty of Nursing, Northern Border University, Arar, SAU; 6 Faculty of Nursing, Northern Border University, Arar, SAU

**Keywords:** nursing students, musculoskeletal pain, musculoskeletal disorders, mobile phone, addiction

## Abstract

Introduction

Smartphones are widely utilized by individuals on a daily basis for a variety of activities, including communication, web browsing, and gaming. However, the excessive and prolonged use of these devices often leads to adverse effects on musculoskeletal health. This study aimed to assess the effect of mobile phone use on musculoskeletal complaints among nursing students at Northern Border University.

Methods

An analytical descriptive study was conducted using a convenience sample of 202 nursing students recruited via a Google survey questionnaire. The questionnaire comprised four sections concerning the students' demographics, smartphone data collection tools, the Smartphone Addiction Scale (SAS-SV), and the Nordic Musculoskeletal Questionnaire (NMQ).

Results

Among the study participants, 62.6% were females, with 52.4% spending more than 5 hours daily on media and technology usage, often extending into bedtime. In the last 12 months, neck pain was the most prevalent complaint, reported by 38.8% of the participants, followed by shoulder pain experienced by 20.3% of the sample. Notably, female students displayed a significantly higher incidence of musculoskeletal pain, with rates as high as 81.7% showing statistical significance (p<0.001). A significant association was also observed between mobile phone addiction and academic grade, as well as the time spent on media and technology usage daily (p<0.001).

Conclusions

There is a significant association between mobile phone use and discomfort in the musculoskeletal system, particularly in the neck region. Moreover, female students tend to experience more pronounced musculoskeletal discomfort compared to their male counterparts. The study also suggests that extended usage of mobile phones, especially at bedtime, increases the likelihood of experiencing musculoskeletal discomfort.

## Introduction

The ubiquitous presence of the internet and electronic devices has revolutionized various aspects of human life globally. As people of all ages actively participate in various online activities, it becomes essential to scrutinize the potential consequences of uncontrolled technology usage, particularly among children and teenagers. While the advantages of internet-enabled devices are evident, ranging from enhanced connectivity to instant access to information, unregulated technology consumption among the youth has raised concerns about possible adverse health impacts [1].

In the present era, smartphones have emerged as a widely prevalent and influential technology, especially among the younger population. These devices have revolutionized daily life by facilitating social networking, gaming, effortless communication, navigation, media consumption, and swift internet access. However, the extensive utilization of smartphones has been associated with an increased risk of musculoskeletal discomfort [2].

Saudi Arabia has witnessed significant socio-economic advancements recently, with notable progress in economic and educational domains [3]. The surging popularity of smartphones among Saudis symbolizes a modern and contemporary way of life. Recent research reveals that most Saudi university students possess smartphones, with an estimated ownership rate exceeding 95%. This exponential rise in smartphone usage has been fueled by the desire to access social media networks, which have become an integral part of daily life [4].

Nursing students extensively utilize mobile phones for various purposes, including accessing medical literature, communicating with fellow students, and utilizing healthcare applications. However, ongoing concerns about the growing reliance on mobile devices may impact nursing students' musculoskeletal health [5].

Cultural perceptions surrounding health and discomfort, including the degree of openness in discussing physical ailments and seeking treatment, may affect the prevalence and reporting of conditions like musculoskeletal pain [6]. Furthermore, regional factors, such as the level of physical activity influenced by the hot climate, the nature of social and leisure activities, and the accessibility and use of healthcare resources, are also crucial considerations [7].

Musculoskeletal challenges encompass various conditions that impact components of the body’s support system, including muscles, bones, tendons, ligaments, and more. The prolonged and repetitive use of mobile phones, coupled with inadequate ergonomics, can potentially lead to muscular and joint problems among nursing students. These issues could be manifested as neck pain, discomfort in the shoulders, upper back pain, and issues in the hands or wrists [8].

Recent evidence suggests a troubling trend known as 'text neck,' a condition arising from the degenerative impact on the cervical spine caused by the repetitive stress of extended periods of forward head flexion while looking down at mobile screens [9]. Without adequate intervention, the progression of text neck can lead to a multitude of physical health complications, including changes in cervical curvature, neck and shoulder muscle strain, compromised neck muscle proprioception, damage to posterior ligaments, and entrapment neuropathies [10]. Associated disorders stemming from a sustained flexed head posture encompass a range including cervicogenic headaches, cervicogenic dizziness, and cervical radiculopathy, all of which present with painful symptoms and functional impairments of the spine. Furthermore, research indicates that cell phones requiring only one thumb or finger for texting increase the likelihood of developing musculoskeletal ailments [11]. The prevalence of musculoskeletal problems among mobile phone users ranges from 1.0% to 67.8%, with neck pain being the most frequently reported issue [12]. The association between mobile device use and musculoskeletal disorders in nursing students was investigated in numerous recent studies. Ogunlana et al. [13] evaluated the prevalence and risk factors of musculoskeletal discomforts among undergraduate students who frequently used their phones. Their findings emphasized the necessity of implementing preventative measures by establishing a significant association between cell phone use and symptoms of musculoskeletal problems. Additionally, Gutiérrez-Puertas V et al. [5] analyzed the association between mobile phone use by nursing students and their musculoskeletal problems, focusing on how these issues affected their day-to-day activities. Also, Hasiholan BP and Susilowati IH [14] identified a significant association between standing and leaning on a table posture and elbow, knee, and ankle complaints in cross-sectional research involving 709 students to determine muscle and bone complaints from mobile phones.

Understanding the impact of mobile phone usage on musculoskeletal symptoms among nursing students is highly important, as these concerns can significantly affect their academic performance, quality of life, and future professional practice [15]. Furthermore, it is essential to explore practical strategies for mitigating potential harm, given the increasing prevalence of mobile phone usage among nursing students [16].

This study aims to explore the association between the usage patterns of mobile phones, ergonomic practices, and the occurrence of musculoskeletal problems in nursing students at our institute. The findings can contribute to advancing therapies and initiatives to enhance musculoskeletal health. By identifying the risk factors associated with cell phone use and musculoskeletal symptoms, this study can help raise awareness and develop effective interventions, preventive strategies, ergonomic interventions, or educational programs to mitigate musculoskeletal complaints associated with mobile phone use among nursing students

## Materials and methods

Research design

A cross-sectional descriptive study was conducted following the “Strengthening the Reporting of Observational Studies in Epidemiology” (STROBE) recommendations [17]. The extent of smartphone addiction was assessed using the Smartphone Addiction Scale Short Version (SAS-SV) [18], while respondents' musculoskeletal discomfort was assessed using the Nordic Musculoskeletal Questionnaire (NMQ) [19].

Participants

This study recruited both male and female nursing students from Northern Border University who owned smartphones, ranged from first to fourth grade, were aged 18 to 23 years, and agreed to participate in the study. The sample consisted of 202 participants, determined based on the total enrollment of 425 nursing students in the College of Nursing. The following equation was applied for sample size calculation to provide a power of 0.80, a type I error [a] of 0.05, a type II error [b] of 0.2, and a confidence level of 95%: n =

$$(N &times;p (1-p))/([[N-1 &times;(d^(2 )&divide; z^(2 ) )]+p (1-p)])$$

 =

$$(425&times;0.50(1-0.50))/⟦[425-1&times;[0.05x^2&divide;1.96 x^2]]+0.50[1-0.50]⟧$$.

A convenience sampling method was used to avoid the risk of low response rates for this study. Although the initial calculated sample size was 202 participants, we subsequently enrolled a total of 227 nursing students who met the inclusion criteria and consented to participate. Including these additional participants not only increased the study's power but also provided a more comprehensive analysis of the impact of mobile phone use on musculoskeletal complaints, ensuring that the findings reflect the experiences of the entire cohort.

Data collection

Data were collected electronically via a self-administered Google survey between September 2023 and October 2023. The researchers shared the links with all students after explaining and clarifying the aim of the study and obtaining consent for participation. The data collection tool consists of four sections: (1) sociodemographic data of the students, including age, sex, marital status, year level, and mobile phone usage; (2) a smartphone data collection tool, which includes questions about the number of hours spent using the smartphone, the extent to which the smartphone affects regular sleep hours, and if the student faces muscle and bone-related problems; (3) a 10-out-of-33-question short version of the "Smartphone Addiction Scale (SAS-SV)," with a smartphone addiction threshold value of 31 points for male respondents and 33 for female respondents, as identified by Kwon et al. [20], to determine addiction status; (4) the "Nordic Musculoskeletal Questionnaire (NMQ)," a tool for determining the presence of musculoskeletal discomfort. The validity and reliability of the NMQ, developed by Kuorinka I et al., have been examined, offering credible and practical information on musculoskeletal pain [21].

Ethical considerations

Ethical approval was obtained from the Local Committee of Bioethics (HAP-09-A-043) at Northern Border University (approval # 74/64/H) on March 09, 2023. Administrative agreement was attained from the dean of the Nursing College. Consent was obtained from participants before their inclusion in the study, after explaining the study's aim. Participants were informed that all information was coded and confidentiality maintained.

Statistical analysis

Data were tabulated using descriptive statistics, with frequencies (percentages) for qualitative variables and means/standard deviations (SD) for quantitative variables. The SPSS version 26 (IBM Corp., Armonk, NY, USA) was used for data analysis. The Shapiro-Wilk and Kolmogorov-Smirnov tests were applied to check the normal distribution of the data. Chi-square test, Fisher's exact test, and Monte Carlo test were applied where appropriate to compare qualitative variables. Binomial logistic regression analysis was used to estimate the odds of musculoskeletal complaints among nursing students as a function of several predictor variables, including demographic characteristics and factors related to mobile phone usage. This method estimates the odds ratio for each independent variable, depicted as the exponentiation of the regression coefficients (Exp(β)), indicating the change in odds of the outcome for a one-unit change in the predictor. Statistically significant differences were identified with p-values <0.05, indicating a 95% confidence level.

## Results

Demographic characteristics of the study population

Table 1 showcases the demographic characteristics of study nursing students. It reveals that most students (81.9%) were below 20, with a mean age of 19.33±1.197. Among the students, females accounted for 62.6%, while 39.6% were in their first grade and 33.5% were in their third grade. All students owned smartphones, and 25.1% also had tablets. More than half of the students (52.4%) spent more than 5 hours daily engaging with media and technology. The usage of digital technology for talking on the phone and chatting was reported by 92.5% and 89.4% of the students, respectively. Unfortunately, most students admitted to using digital technology at bedtime, with 48.6% using it for less than two hours and 34.7% using it for 2 to 3 hours before bedtime.

**Table 1:** Distribution of students regarding the personal characteristics (n = 227). Data are presented as numbers (No.) and percentages (%). *: Response not mutually exclusive.

Items	Category	No. (%)
Age/ years	≤20	186 (81.9%)
	>20	41 (18.1%)
	Mean±SD	19.33±1.197
Sex	Male	85 (37.4%)
	Female	142 (62.6%)
Grade	First-year students	90 (39.6%)
	Second-year students	33 (14.5%)
	Third-year students	76 (33.5%)
	Fourth-year students	28 (12.3%)
Digital technology ownership*	Smartphone	227 (100%)
	Desktop computer	14 (6.2%)
	Tablet	57 (25.1%)
	Laptop	31 (13.7%)
Time spent daily on media and technology usage	Less than 2 hours	6 (2.6%)
	2 to 3 hours	38 (16.7%)
	4 to 5 hours	64 (28.2%)
	More than 5 hours	119 (52.4%)
Uses of digital technology that you do on your device*	Playing video games	108 (47.6%)
	Talking on the telephone	210 (92.5%)
	Chatting	203 (89.4%)
	Internet browsing	172 (75.8%)
Technology usage at bedtime	Yes	173 (76.2%)
	No	54 (23.8%)
Hours spent using technology at bedtime (n=173)	Less than 2 hours	84 (48.6%)
	2 to 3 hours	60 (34.7%)
	4 to 5 hours	15 (8.7%)
	More than 5 hours	14 (8.1%)

Nursing student response regarding mobile phone addiction

Analysis of the students' responses to the mobile phone addiction questionnaire (Table 2) estimates that a notable proportion (28.2%) were addicted to mobile phone usage.

**Table 2:** Nursing student response regarding reported mobile phone addiction using Smartphone Addiction Scale Short Version (SAS-SV) (n = 227).

Smartphone Addiction Scale (SAS-SV) Parameters	Mean ± SD
“Missing planned work due to smartphone use”	2.44 ± 1.18
"Difficulty concentrating in class or while working due to smartphone use"	2.69 ± 1.19
"Pain in wrists or back of the neck while using a smartphone"	2.60 ± 1.21
"Inability to stand not having a smartphone"	3.31 ± 1.26
"Impatience and fretfulness when not holding my smartphone"	2.39 ± 1.19
Constantly thinking about my smartphone even when not using it"	2.69 ± 1.19
"Unwillingness to give up smartphone use despite daily life impact"	2.65 ± 1.15
"Frequent checking of smartphone to avoid missing conversations on social media"	2.86 ± 1.24
"Using smartphone for longer durations than intended"	3.19 ± 1.18
"People around me telling me I use my smartphone excessively"	2.74 ± 1.25
Total Smartphone addiction	2.76 ± 0.83

Prevalence of musculoskeletal pain and distribution among nursing students

The study found a high incidence of musculoskeletal pain among nursing students, with 71.4% reporting such discomfort. Neck pain emerged as the most significant concern, with the highest prevalence reported in the last 12 months (38.8%) and the last seven days (20.3%). In contrast, pain in the ankles/feet was the least reported over the past year (3.5%), with no occurrences noted in the previous week. For a detailed breakdown of musculoskeletal pain prevalence across various body parts in both time frames (Table 3).

**Table 3:** Distribution of students according to students’ reported musculoskeletal pain (n = 227). Data are presented as numbers (No.) and percentages (%).

Musculoskeletal Parameters	Pain (12 M)		Pain [7 days)		Both	
	No.	%	No.	%	No.	%
Neck	88 (38.8%)		46 (20.3%)		37 16.3%	
Shoulders	60 (26.4%)		32 (14.1%)		23 (10.1%)	
Elbows	22 (9.7%)		17 (7.5%)		11 (4.8%)	
Wrists/hands	25 (11.0%)		10 (4.4%)		8 (3.5%)	
Upper Back	42 (18.5%)		24 (10.6%)		16 (7.0%)	
Low back	52 (22.9%)		30 (13.2%)		20 (8.8%)	
Hips/thighs	21 (99.3%)		12 (5.3%)		8 (3.5%)	
knees	19 (8.4%)		10 (4.4%)		7 (3.1%)	
Ankles/ Feet	8 (3.5%)		0 (0%)		0 (0%)	

Relation between study student characteristics and musculoskeletal pain

The relationship between nursing student characteristics and musculoskeletal pain is illustrated in Table 4. It shows that female students (81.7%) experienced musculoskeletal pain with a significant difference (p < 0.001). Furthermore, students older than 20 years (87.8%), fourth-year students (92.9%), students who spend more than 5 hours daily on media and technology usage (83.3%), and students who use technology at bedtime (78.3%) experienced musculoskeletal pain, with significant differences (p < 0.05).

**Table 4:** Relation between study's student characteristics and musculoskeletal pain (n = 227). Data are presented as numbers (No.) and percentages (%). A chi-square and Fisher's exact tests were applied when appropriate. Statistical significance was set at p-values less than 0.05. * p< 0.05, ** p< 0.001.

Students' characteristics	Category	Musculoskeletal pain		p-value
		Pain	No pain	
		N (%)	N (%)	
Age/ years	≤20	126 (67.7%)	60 (23.3%)	0.010*
	>20	36 (87.8%)	5 (12.2%)	
Sex	Male	46 (54.1%)	39 (45.9%)	0.000**
	Female	116 (81.7%)	26 (18.3%)	
Grade	First-year students	58 (64.4%)	32 (35.6%)	0.036*
	Second-year students	23 (69.7%)	10 (30.3%)	
	Third-year students	55 (72.4%)	21 (27.6%)	
	Fourth-year students	26 (92.9%)	2 (7.1%)	
Time spent daily on media and technology usage	Less than 2 hours	5 (83.3%)	1 (16.7%)	0.018*
	2 to 3 hours	20 (52.6%)	18 (47.4%)	
	4 to 5 hours	42 (65.6%)	22 (34.4%)	
	More than 5 hours	95 (79.8%)	24 (20.2%)	
Using technology at bedtime	Yes	112 (78.3%)	31 (21.7%)	0.002*
	No	50 (59.5%)	34 (40.5%)	

Relation between study student characteristics and mobile phone addiction

Significant differences were observed in mobile phone addiction based on grade level, daily time spent on media/technology usage, and technology usage at bedtime (Table 5). Among second-grade students, the highest percentage of mobile phone addiction was observed at 54.5%. Students who spend more than five hours daily on media and technology usage had the highest percentage of mobile phone addiction at 40.3%, and using technology at bedtime was associated with a notable percentage of mobile phone addiction among students, amounting to 35%.

**Table 5:** Relation between study's student characteristics and mobile phone addiction (n = 227). Data are presented as numbers (No.) and percentages (%). A chi-square and Fisher's exact tests were applied appropriately. Statistical significance was set at p-values less than 0.05. * p< 0.05, ** p< 0.001.

Students' characteristics		Mobile phone addiction		p-value
		Addict	Not addict	
		N (%)	N (%)	
Age/ years	≤20	52 (28%)	13 (72%)	0.866
	>20	12 (29.3%)	29 (70.7%)	
Sex	Male	26 (30.6%)	59 (69.4%)	0.535
	Female	38 (26.8	104 (73.2%)	
Grade	First-year students	12 (13.3%)	78 (86.7%)	0.000**
	Second-year students	18 (54.5%)	15 (45.5%)	
	Third-year students	26 (34.2%)	50 (65.8%)	
	Fourth-year students	8 (28.6%)	20 (71.4%)	
Time spent daily on media and technology usage	Less than 2 hours	1 (16.7%)	5 (83.3%)	0.000**
	2 to 3 hours	4 (10.5%)	34 (89.5%)	
	4 to 5 hours	11 (17.2%)	53 (82.8%)	
	More than 5 hours	48 (40.3%)	71 (59.7%)	
Using technology at bedtime	Yes	50 (35%)	93 (65%)	0.004*
	No	70 (83.3%)	14 (16.7%)	

The relationship between musculoskeletal pain, students' characteristics, and mobile phone addiction

As depicted in Table 6, the coefficient for age (β=0.374) suggests that with each additional year, the odds of experiencing musculoskeletal pain increase by a factor of 1.4, although this finding was not statistically significant (p=0.196). However, the analysis revealed a significant relationship between sex and the presence of musculoskeletal pain, with a coefficient of β=1.327. This indicates that females are 3.7 times more likely to report musculoskeletal pain than males, which is significant (p<0.001).

**Table 6:** The binomial logistic regression analysis of the studied variables. The dependent variable is the musculoskeletal pain, and the independent variables are the students' characteristics and mobile phone addiction. Statistical significance was set at p-values less than 0.05. * p< 0.05, ** p< 0.001. β (Beta): The regression coefficients; SE: The standard error of the regression coefficient; Wald: Wald Statistic used to test the null hypothesis; Sig.: Significance; Exp(β): Exponential function of Beta. The constant (intercept) represents the log odds of the outcome when all independent variables are zero.

Items	b	SE	Wald	Sig.	Exp(b)
Age/ years	0.374	0.289	1.675	0.196	1.453
Sex	1.327	0.352	14.212	0.000**	3.769
Grade			4.709	0.194	
First-year students	0.250	1.169	0.046	0.831	1.284
Second-year students	-0.912	1.010	0.814	0.367	0.402
Third-year students	-0.536	0.856	0.392	0.531	0.585
Time spent daily on media and technology usage	0.156	0.219	0.512	0.474	1.169
Using technology at bedtime	0.436	0.362	1.452	0.228	1.546
Mobile phone addiction score	0.062	0.025	6.178	0.013*	1.064
Constant	-10.527	6.463	2.653	0.103	0.000

While the overall effect of academic grade level was observed (Wald=4.709, p=0.194), individual coefficients for each grade did not reach statistical significance. This suggests that, although there appears to be a pattern, there is insufficient evidence to conclude a definitive grade-level impact on musculoskeletal pain in this sample. Interestingly, a significant relationship was found between the mobile phone addiction score and musculoskeletal pain (β=0.062, p=0.013).

## Discussion

The widespread adoption of mobile phones in recent years raises the possibility that musculoskeletal diseases may severely impact nursing students. This study aimed to determine how nursing students at the authors' institute were affected by their usage of mobile phones in terms of musculoskeletal complaints.

The present study indicates that most participants were below 20 years old, with a mean age of 19.33±1.197. More than two-thirds of the participants were females, with over one-third in the first and third academic grades. All participants owned smartphones, while a quarter also had tablets. In the study “Impact of Digital Device Use on Neck and Low Back Pain Intensity among Nursing Students at a Saudi Government University” by Mahmoud NA et al. [22], it was found that more than half of the respondents were under 22 years old, with an average age of 21.4±1.7 years. Furthermore, more than four-fifths of the respondents in that study were female, and more than one-quarter had progressed to the seventh academic level. Our figures align with the general demographic profile of nursing students in the region [23,24]. Another survey conducted in Jeddah, Saudi Arabia, involving 428 medical students from six medical colleges, revealed that more than three-fifths of participants were female. The average age of the participants in that survey was 22.11±2.07 years, with more than one-quarter being fourth-year students [25]. It is worth noting that age-related anatomical and physiological characteristics may influence the prevalence or reporting of musculoskeletal issues. We acknowledge that musculoskeletal development during late adolescence and early adulthood is critical, particularly as the nursing profession includes physically demanding tasks that might exacerbate underlying pediatric anatomical variations [10]. The potential for both an increased risk of musculoskeletal symptoms due to the vulnerability of developing structures and the capacity for a higher threshold for physical stress due to youthful resilience should be considered.

In the present study, we observed substantial daily use of media and digital technology, primarily for communication, with a notable trend of device usage extending to bedtime. These patterns might be partially ascribed to the Saudi Arabian climate, which encourages indoor activities during the hot seasons, and certain sociocultural norms that subtly influence entertainment choices and social interactions, particularly for women. This propensity is further supported by the findings of Mahmoud NA et al. [22], who noted a pervasive daily engagement with digital devices among participants, a trend that starts from an early age, often before thirteen. However, it is crucial to recognize that this behavior is not uniform across the entire population, as variations in lifestyle, access to digital technology, and personal preferences significantly influence individual habits. While our data suggest a link between environmental and societal factors and increased screen time, this should be viewed in parallel with global digital trends that transcend regional specifics.

Fortunately, a recent study by Maayah MF et al. [4] in Saudi Arabia found that individuals used smartphones daily, with an average frequency of 798 times per week, representing 92.0% of all days. However, there were instances where the lowest frequency recorded was only 20 times, accounting for 2.3% of 1-2 day periods. Regarding the average number of hours spent on smartphones daily, the study showed that the highest percentage spent equal to or more than 4 hours, while the lowest percentage spent less than 4 hours.

tatement 'Will not be able to stand not having a smartphone' had the highest average score (3.31±1.259), indicating a significant addiction to smartphones among most students, who would feel worried or uncomfortable without it. 'Feeling impatient and fretful when I am not holding my smartphone' had the lowest average score (2.39±1.2), suggesting that most students do not experience significant emotional discomfort without smartphones. The overall smartphone addiction average score was 2.76±0.8, indicating moderate addiction among the students. As demonstrated in Ahmed S et al.'s study, more than three-fifths of the 326 participants were moderately to severely addicted to their cell phones [26]. In contrast, prior research from Ordu University in Turkey found only a slight incidence of smartphone addiction, the mean score was 25.71±7.49 [27].

Based on several studies, smartphone addiction harms both physical and mental health. Regarding the relationship between musculoskeletal issues on the one hand and smartphone addiction and dependence on the other, there have been concerns [28,29].

In previous studies conducted among 516 university students in Saudi Arabia, it was found that electronic devices were widely used by the participants, with the majority of them utilizing these devices. Among these users, more than four-fifths utilized smartphones and iPads specifically for entertainment, while less than one-third utilized them for academic activities. Additionally, approximately three-fifths of the participants reported experiencing neck or shoulder pain using electronic devices [24].

Regarding musculoskeletal pain among nursing students, our study revealed that the neck was the most consistently affected area in the last 12 months and the last seven days, though only a minority of participants reported pain. Conversely, the wrists/hands, hips/thighs, knees, and ankles/feet had the lowest prevalence of pain. These findings may result from prolonged periods of sitting or inadequate ergonomics during clinical practice or study sessions and incorrect mobile phone use. These findings align with a study by Alsalameh AM et al. [28], where the authors found that the neck and lower back experienced the most pain related to smartphone addiction. Similarly, previous research by Mahmoud NA et al. on nursing students at a Saudi government university reported that nearly half of the participants experienced minor neck discomfort [22]. Overall, these findings highlight the prevalence of musculoskeletal pain among nursing students, with the neck being the most commonly affected area. A comprehensive review of 15 articles indicated that neck-related musculoskeletal problems were the most common among mobile device users (17.3-67.8%) [30].

Similarly, there is a clear link between neck discomfort and smartphone addiction, as explained in several studies [28,31,32]. In a recent study conducted by Walankar PP et al. [11], it was found that out of 2000 students, more than two-fifths reported experiencing musculoskeletal discomfort. The most commonly affected areas included the neck, thumb, lower back, and elbow. This study highlights the importance for students to be mindful of the duration for which they use their smartphones, as it directly impacts the occurrence of musculoskeletal problems.

Concerning the relationship between nursing student characteristics and musculoskeletal pain, the findings illustrated that most female students experienced pain, showing significant differences. These findings may be attributed to Saudi Arabia's society, which has traditional gender roles and expectations, where females may be more likely to engage in activities contributing to musculoskeletal pain, and female students may spend more time on mobile phones. Additionally, cultural norms may present further challenges for females. Also, most students spend more than 5 hours daily on media and technology usage, and more than three-quarters of students who use technology at bedtime experience musculoskeletal pain. In the study conducted by Ayar D and Gürkan KP, it was found that more than three-fifths of nursing students were females, and nearly two-fifths of all participants spent more than five hours daily on the internet. Upon examining the reasons for their internet usage, it was revealed that a small percentage engaged in online gaming, while a considerable majority used the internet for various other purposes [33].

The present study's findings revealed a significant difference in mobile phone addiction based on academic grade level, with the highest proportion observed among second-grade students. It also demonstrated that the amount of time spent daily on media and technology usage is associated with mobile phone addiction, as students who spend more than five hours daily on these activities had the highest percentage of addiction. Furthermore, the study found a notable difference in mobile phone addiction based on the use of technology at bedtime, with more than one-third of students reporting this behavior. These results align with the findings of Farghaly Abdelaliem SM et al. [34], who discovered a highly significant correlation between smartphone addiction and students' age, academic level, and specialty. However, it contradicts the findings of Machado J et al., who found no substantial relation between gender, year of study, location of stay, and smartphone addiction [35]. The contrasting outcomes might be attributed to the variations in the research, where the investigators specifically targeted female Indian nursing students in their third and fourth years. This demographic exclusivity, along with the predominance of female participants comprising the majority of the study sample, could explain the conflicting findings.

These findings are in line with the research conducted by Maayah MF et al. [4], which supports the hypothesis that college students who spend increased time on their cell phones and have a history of neck discomfort are more prone to developing chronic neck pain. Consequently, we suggest that students should consider limiting their cellphone usage.

Furthermore, research by Hanphitakphong P et al. explored significant connections between upper-body musculoskeletal complaints and smartphone addiction, gender, and age. Using multiple logistic regression analysis, the researchers identified female gender, smartphone addiction, and students over 20 as predictors for greater upper-body musculoskeletal complaints [36].

Results of the present study indicated that age, grade, time spent daily on media/technology usage, and using technology at bedtime were not significant predictors of musculoskeletal pain. As stated by Khalid A et al. [37], there was no significant relationship between age and the modified neck and shoulder disability index, nor was there any relationship between gender and the modified neck and shoulder disability index. Additionally, a previous study conducted on 120 nursing students at a university in Riyadh, the capital city of Saudi Arabia, found no significant relationship between neck pain and either age or gender [22].

Study limitations

This study on mobile phone use among nursing students has several limitations, including limited generalizability due to its focus on a specific university population, potential response bias due to self-reported data, and the cross-sectional design that only captures data at a single point in time. Additionally, using a convenience sampling method might have impacted the representativeness of our sample, as participants were selected based on availability rather than through a random process. There is also the absence of objective measures to quantify mobile phone use and validate musculoskeletal complaints. Large-scale and longitudinal studies could provide more robust evidence for investigating the long-term effects of mobile phone use on musculoskeletal complaints.

Despite the limitations mentioned above, we believe our work offers the following original contributions: (1) Focused Exploration Within a Distinct Population: Our research specifically targets nursing students, who may experience unique stressors related to their rigorous training and clinical responsibilities. This focus presents new data on the intersection of mobile technology use and physical health in this demographic; (2) Contextual Application of Findings: The insights gleaned from our study are not only relevant to the health and academic performance of nursing students but may also have implications for their future professional practice, given the necessity of maintaining musculoskeletal health in a healthcare setting; and (3) Basis for Future Interventions: By identifying correlations within this particular group, we provide a foundation for educational institutions to develop targeted ergonomic and health promotion interventions to prevent musculoskeletal complaints among students preparing for physically demanding healthcare professions.

## Conclusions

The present results indicate a significant association between mobile phone use and musculoskeletal discomfort. The findings conclude that prolonged and repetitive use of mobile devices, such as smartphones and tablets, can cause musculoskeletal pain and discomfort, especially in the neck, shoulders, and upper back areas. Additionally, gender variations were identified, demonstrating that female students suffer from more significant musculoskeletal discomfort than male students. Also, prolonged use of mobile phones, especially at bedtime, makes them more prone to musculoskeletal discomfort.

Based on the findings, it is recommended that students take a break every 30 minutes to stretch or change their position. Recommendations for reducing discomfort caused by extended mobile phone use include incorporating exercises that target the muscles used while using a phone, such as neck stretches, shoulder rolls, and upper back exercises. These exercises aim to enhance muscular strength and reduce the likelihood of developing musculoskeletal discomfort. Additionally, it is crucial to raise students' awareness about the potential risks associated with prolonged mobile phone use and offer guidance on maintaining good posture and ergonomic practices while using these devices.
